# Book Reviews

**Published:** 1899-11

**Authors:** 


International Clinics. A Quarterly of Clinical Lectures on Medicine, Neurology, Surgery, Gynecology and Obstetrics, Ophthalmology, Laryngology, Pharyngology, Rhinology, Otology and Dermatology, and Specially Prepared Articles on Treatment and Drugs. By professors and lecturers in the leading medical colleges of the United States, Germany, Austria, France, Great Britain and Canada. Edited by Judson Daland, M. D. (Univ. of Penna.), Philadelphia, Instructor in Clinical Medicine and Lecturer on Physical Diagnosis in the University of Pennsylvania; Assistant Physician to the Hospital of the University of Pennsylvania, etc.; Fellow of the College of Physicians of Philadelphia. Volume II. Ninth series. 1899. Octavo, pp. x.—310. Philadelphia: J. B. Lippincott Co. 1899.

Among the present contributors to this volume at home are William Cheatham, Charles Green Cumston, Henry L. Elsner, Horace H. Grant, Hobart Amory Hare, W. W. Keen and Henry M. Lyman; and abroad are, L. Brocq, A. Fournier, Fränkel, Ernst Grawitz, Gutzman, Jaccoud, König, Lassar and von Bergman. The topics embraced pertain to drugs and remedial agents, treatment, medicine, neurology, surgery, gynecology and obstetrics, ophthmology, laryngology and dermatology. There are a few good illustrations, but as we have repeatedly pointed out, too little attention is paid to this important feature. If more care were taken in the preparation of some of the lectures that appear in the International Clinics, as well as a more complete attempt at illustrating many of them, additional interest would be stimulated regarding this most excellent plan for diffusing the latest and best of medical literature.

The fact is, it will not answer to trust so important a matter as the reporting of a clinical lecture to an inexperienced or indifferent medical student. The greatest care should be taken by the lecturer to present excellent material, and to see that it is properly brought before the reader. This requires careful editing of the report, and excellent proof-reading as well.

These are general ideas we present for the consideration of those most interested, and have no special relation to the book before us. It is a most excellent volume, fully up to its predecessors, and makes a valuable addition to the series.

---

Surgical Nursing. A Compilation of the Lectures upon Abdominal Surgery, Gynecology and General Surgical Conditions and Procedures delivered to the classes in the training school for nurses connected with the Woman's Hospital of Philadelphia. By Anna M. Fullerton, M. D., Clinical Professor of Gynecology in the Woman's Medical College; Obstetrician, Gynecologist and Surgeon to the Woman's Hospital. Third edition, revised and enlarged, with sixty-nine illustrations. Philadelphia: P. Blakiston's Son & Co. 1899.

This book is issued under a new name and is improved very much. The former title, Nursing in abdominal surgery and diseases of women, restricted its scope; whereas, under the broader name, Surgical nursing, the other subjects are included and the field covered by the book is expanded.

Dr. Fullerton always writes interestingly, and she has not omitted to do so in this effort. She is a teacher of experience and knows how to utilise her observations to the best advantage. A considerable portion of this book is devoted to the principles which govern aseptic wound surgery—a subject of great importance to nurses. They should be well trained in the doctrine of cleanliness.

---

A Text-Book of Pharmacology and Therapeutics, or the Action of Drugs in Health and Disease. For the use of students and practitioners of medicine. By Arthur R. Cushny, M. A., M. D., Aberd., Professor of Materia Medica and Therapeutics in the University of Michigan Medical Department, Ann Arbor. In one octavo volume of 728 pages, with forty-seven engravings. Philadelphia and New York: Lea Brothers & Co. 1899.

The literature of therapeutics is further enriched by this work of Cushny, which embraces, as its title indicates, pharmacology. What the student most desires is a knowledge of the scientific and efficacious use of drugs—that employment of them that will result most frequently in cure. There has been a wide gap between the lecture room and the clinic on this subject and this author endeavors to supply the missing link, so to speak.

He treats the subject from the experimental viewpoint, which, in the present period, is the only rational way to handle it. Drugs and pharmaceutical preparations that do not stand the test of the clinic must step aside, and only those that meet this critical method can remain. Only such are entitled to the confidence of the scientific physician.

Cushny discusses the action of drugs with logical fairness, and gives a student a pretty clear insight of this important branch of his subject in the early pages of the book He then divides his treatise into six parts. Part I. considers organic substances, which are chiefly by their local action; Part II. deals with organic substances, characterised chiefly by their action after absorption; Part III. treats of combination of the alkalies, alkaline earths, acids and allied bodies; Part IV. handles the heavy metals; Part V. takes up ferments, secretions and toxalbumins, while part VI. presents menstrua—and mechanical remedies.

This classification is excellent and Cushny's method of dealing with each is intelligent. We have no doubt the book will obtain the confidence of teachers and become a standard work in the class room.

---

PRACTICAL DIAGNOSIS. The Use of Symptoms in the Diagnosis of Disease. By HOBART AMORY ARE, M. D., B. Sc., Professor of Therapeutics and Materia Medica in the Jefferson Medical College of Philadelphia. Fourth edition, enlarged and thoroughly revised. Octavo, pp. 623, with 205 engravings and fourteen full page colored plates. Philadelphia and New York: Lea Brothers & Co. 1899.

For the second time within a year we are invited to write a notice of separate editions of this treatise, the third having appeared in the autumn of 1898, and this, the fourth, in the summer of 1899. This is only more remarkable than the fact that the first edition of this work was sent out in August, 1896, thus making four in a little less than three years. Nothing could be more gratifying, we imagine, to the distinguished author, than this marked appreciation of the value of his writing, or such a precious testimonial to the merits of the products of his pen.

We have heretofore, as the several editions issued from the press, expressed our views upon the subject matter which they have contained, and we are prepared, upon a reëxamination of both books and reviews, to reaffirm our former opinions so frequently set forth as to the usefulness of this work to student, practitioner and teacher. It is essentially a clinical treatise on diagnosis, which is the only proper standpoint from which to write such an important part of the foundation of proper medical teaching. From the bedside must come the language of disease and thence must emanate all the artful knowledge pertaining to the interpretation of symptoms.

As a matter of course, since the third edition of this book issued from the press last fall, very little that is new has been invented or discovered with reference to the diagnosis of disease, hence this volume cannot possess many advantages over its immediate predecessor. However, taking advantage of the opportunity, the author has made some revisions of the text and added a few illustrations, including a colored plate.

There is small need in saying that up to the present time no treatise on the subject excels Hare's, and none exceed it in popularity. This is due to the method chosen by the author in the presentation of his subject, in its clear and concise verbiage, and in its scientific tone from cover to cover.

ELECTRO-HEMOSTASIS IN OPERATIVE SURGERY. Being a Work Supplementary to the Author's Treatise on the Diseases of Women. By ALEXANDER J. C. SKENE, M. D., LL. D., Professor of Gynecology in the Long Island College Hospital, Brooklyn; Gynecologist to the Long Island College Hospital, etc., etc. Octavo, pp. x.—173. Illustrated. New York: D. Appleton & Co. 1899.

The author of this book is a well-known contributor to the literature of gynecological medicine as well as a surgeon of ability who has met with almost every complication that can arise in surgical practice. The present work is issued as a supplement to the third edition of the author's treatise on diseases of women, wherein this subject was only briefly referred to. This monograph amplifies and develops the subject into a broader discussion than would be appropriate in a general treatise.

The most important question, next to the operation itself, is hemostasis. How many patients have been lost because of imperfect intraäbdominal or intrapelvic control of bleeding points it would be impossible to say. Formerly, it was the habit of many, if not all, surgeons to depend upon the *en masse* ligatures, and other incompletenesses in technic, that were responsible for secondary hemorrhages. Nowadays, however, it is the custom to ligate each vessel separately, and even all points that bleed anyway freely are secured with fine ligatures of some sort. The question of disposal of the ligatures, especially those placed to secure large vessels, has been an interesting one that has not yet been fully or satisfactorily settled. These and many more parts of moment arise in considering the methods of hemostasis.

In the ingenious application of electricity to the end in view—namely, an effectual, aseptic, safe way of securing vessels during operations within the cavities of the body. Dr. Skene has added another chaplet to his laurels, and increased the surgical armament most beneficially. Briefly, he treats the vessels with a clamp or forceps, charged with electrical currents in such a manner as to dessicate the tissue, but not to disintegrate it. It becomes therefore impossible for it to bleed, nor does it decay and set up a focus of infection. The author at the time this book was written, had employed the method more than 200 times in abdominal operations, as well as in many vaginal hysterectomies and other operations through the vagina, without having seen secondary hemorrhage after any of them.

A number of cunningly devised instruments are described, which facilitate the application of this method, and make it available in almost every nook and cranny within the abdomen, or elsewhere; indeed, wherever a bleeding vessel may lurk. These are illustrated by numerous engravings, half-tone and wood, which are all described in clear and intelligible language.

The author writes of a subject with which he is familiar, and supports his statements with clinical facts. The book is a most interesting one from beginning to end and should be in possession of every surgeon.

---

Transactions of the Medical Society of the State of New York, for the year 1899. Edited by Frederic C. Curtis, M. D., Secretary. Philadelphia: Wm. J. Dornan, Printer. 1899.

This volume contains forty-nine scientific papers, besides the address of the president, the subject of which is The relation of medicine to circulation, and two special addresses—one by William Osler, entitled The problem of typhoid fever in the United States, and the other by Abraham Jacobi, on The disinfection of the alimentary canal. In addition it contains the usual reports of committees, officers, lists of members of county societies, besides many other matters of importance, not only to members of the profession residing in the Empire State, but to medical men throughout the country.

The president's address, by Dr. John O. Roe, of Rochester, is a scholarly paper, exhibiting a rare knowledge of the history of medicine and a deep interest in its progressiveness as a science. It should be read by all physicians and other scientific men. The addresses by Drs. Jacobi and Osler are full of practical information upon important subjects, and we need scarcely add, are handled both scientifically and interestingly.

The other papers are, for the most part, of a high order, carrying the subjects with which they deal well to the fore. The volume as a whole is one of exceptional interest, exhibiting the work of a well-disciplined medical organisation that was presided over with skill and discrimination, and that has been presented by an experienced editor.

INDEX-CATALOGUE OF THE LIBRARY OF THE SURGEON-GENERAL'S OFFICE, UNITED STATES ARMY. Authors and subjects. Second series, Vol. IV. D—Emulsions. Prepared under the direction of the Surgeon-General by JAMES C. MERRILL, Major and Surgeon U. S. Army, Librarian Surgeon-General's Office. Washington: Government Printing Office. 1899.

This great catalogue, now so formidable as to almost appall the ordinary searcher for medical literature, grows apace with the advancing years. The addition for the present year contains 9,628 author's titles, representing 4,133 volumes and 8,523 pamphlets. It also contains 8,828 subject-titles of separate books and pamphlets, as well as 28,316 titles of articles in periodicals.

The library now contains 130,708 bound volumes and 220,839 pamphlets. The total number of titles contained in the catalogue, as far as published is:

Author-titles—Titles, 219,182; volumes, 107,179; pamphlets, 191,846.

Subject-titles—book-titles, 201,679; journal articles, 625,851; portraits, 5,012.

The gratitude of the medical profession is due to the Surgeon-General of the Army and his colaborers for the splendid manner in which all this vast material is rendered available.

Atlas on Fractures and Dislocations. By Prof. Dr. H. Helferich, of Greifswald. Translated from the third German edition by J. Hutchinson, Jr., F. R. C. S. Sixty-eight chromo-lithographic plates with descriptions and 130 pages of treatise, illustrated by 126 woodcuts. Wood's Series of Medical Hand Atlases. New York: Wm. Wood & Co. 1899.

This book deals with a most vexed question in surgery. Fractures and dislocations have always, and no doubt will for all time to come, present difficulties that only great experience, thorough training and mature judgment may hope to surmount. We speak, of course, of the complications that are liable to arise in delayed, or unrecognised cases, rather than the recent simple ones.

This volume is constructed upon lines that are calculated to aid in the solution of many difficult problems these injuries are liable to present. The colored plates are most telling pictures of actual lesions in the continuity of bone or the integrity of joint.

The author is a surgeon of experience and has illustrated or explained his drawings with clinical notes, printed in clear text. This edition has brought the subject forward to the present period and deserves extended and liberal sale.

---

Transactions of the Twentieth Annual Meeting of the American Laryngological Association. Held in the City of Brooklyn, May 16, 17 and 18, 1898. Henry L. Swain, M. D., Secretary. New York: D. Appleton & Co. 1899.

This excellent special society presents yearly a volume that records scientific work of a most valuable and instructive character. Moreover, it is done in a business-like manner, without flourish of trumpets or other noisy exploitations to attract the passing throng.

The papers printed in the volume constitute a distinct addition to the literature of laryngology, and it should be found on the bookshelves of every physician who devotes himself to the practice of this specialty, or who treats the nose, throat or ear even in a tentative manner.

The membership of the association is made up of some of the most distinguished men in this country, who are identified with the practice of laryngology, hence what they write or say carries great weight. Numerous illustrations are printed in the text, which serve to elucidate it.

